# Generic, network schema agnostic sparse tensor factorization for single-pass clustering of heterogeneous information networks

**DOI:** 10.1371/journal.pone.0172323

**Published:** 2017-02-28

**Authors:** Jibing Wu, Qinggang Meng, Su Deng, Hongbin Huang, Yahui Wu, Atta Badii

**Affiliations:** 1 Science and Technology on Information System Engineering Laboratory, National University of Defense Technology, ChangSha, Hunan, China; 2 Department of Computer Science, Loughborough University, Loughborough, United Kingdom; 3 Department of Computer Science, University of Reading, Whiteknights, United Kingdom; Tianjin University, CHINA

## Abstract

Heterogeneous information networks (e.g. bibliographic networks and social media networks) that consist of multiple interconnected objects are ubiquitous. Clustering analysis is an effective method to understand the semantic information and interpretable structure of the heterogeneous information networks, and it has attracted the attention of many researchers in recent years. However, most studies assume that heterogeneous information networks usually follow some simple schemas, such as bi-typed networks or star network schema, and they can only cluster one type of object in the network each time. In this paper, a novel clustering framework is proposed based on sparse tensor factorization for heterogeneous information networks, which can cluster multiple types of objects simultaneously in a single pass without any network schema information. The types of objects and the relations between them in the heterogeneous information networks are modeled as a sparse tensor. The clustering issue is modeled as an optimization problem, which is similar to the well-known Tucker decomposition. Then, an Alternating Least Squares (ALS) algorithm and a feasible initialization method are proposed to solve the optimization problem. Based on the tensor factorization, we simultaneously partition different types of objects into different clusters. The experimental results on both synthetic and real-world datasets have demonstrated that our proposed clustering framework, STFClus, can model heterogeneous information networks efficiently and can outperform state-of-the-art clustering algorithms as a generally applicable single-pass clustering method for heterogeneous network which is network schema agnostic.

## Introduction

Information networks are widely used to describe realistic applications in the cyber domain. Vertices in information networks map the objects in real-world applications, and edges map the relations between them. While the mining of information networks has been studied for many years, most current studies have focused on homogeneous information networks [1], consisting of only one type of vertex and one type of edge between vertices. For example, the well-known PageRank algorithm [2] models the Internet as a homogeneous information network. Each webpage is mapped to a vertex and each hyperlink between webpages is mapped to an edge.

**Fig 1:**
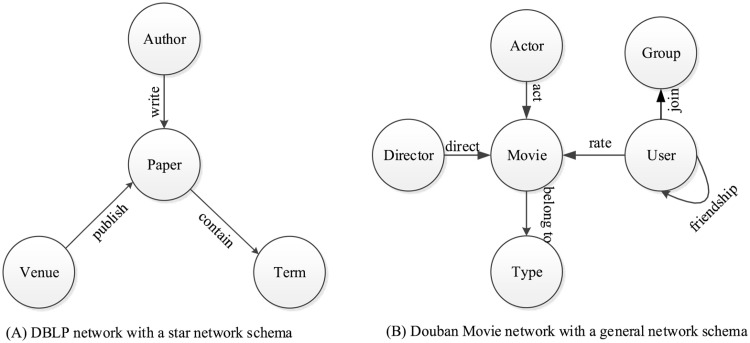
Examples of network schemas for two different heterogeneous information networks. (A): DBLP network with a star network schema. (B): Douban Movie network with a general network schema.

However, in real-world applications, information networks are often heterogeneous, where objects and the relations between them are of more than one type. We call this kind of information network a **heterogeneous information network** [3]. For example, the bibliographic network extracted from the DBLP database (http://dblp.uni-trier.de/db/) is a typical heterogeneous information network, which is shown in Fig 1A. The DBLP database is an open resource that contains most of the bibliographic information on computer science. The network contains four types of objects: author (A), paper (P), venue (i.e., conference or journal) (V), and term (T). The concept of mining heterogeneous information network was first proposed by Y. Sun and J. Han [1].

Clustering is an effective method for understanding the semantic information and interpretable structure of a network. Clustering can also support relation prediction in information networks. Unfortunately, clustering heterogeneous information networks is more difficult than doing so for homogeneous information networks. We cannot directly measure the similarity among the different types of objects and relations. In recent years, researchers have made significant progress in clustering heterogeneous information networks, which largely focuses on the following three main directions.

The first is to use a ranking based clustering algorithm [1], this developed the RankClus algorithm that integrated clustering with ranking for clustering bi-typed networks, where only two different types of objects exist in the network. Its extension, the NetClus [3] algorithm, was developed for the star network schema, where the edges only appear between target objects and attribute objects. Fig 1A shows a typical star network schema, where the paper (P) is the target object and others are attribute objects. RankClus and NetClus have shown that ranking and clustering can mutually enhance each other. The recent work FctClus [4] achieved a higher computational speed and had a greater clustering accuracy when applied to heterogeneous information networks. However as with NetClus, the FctClus algorithm can only handle the star network schema. The network schema is a meta template of a heterogeneous information network, which shows how many types of objects and links in the network. The definition of network schema can be found in [5].

The second direction involves meta-path based clustering algorithms. A meta-path [5] is a connected path defined on the network schema of a heterogeneous information network, which represents a composite semantic relation between two objects. PathSim [5] (Meta-path based top-k similarity search) measured the similarity between the same types of objects based on meta-path in heterogeneous information networks. However, it has a limitation in that the meta-path must be symmetric, i.e., PathSim couldn’t work on different types of objects. The PathSelClus algorithm [6–8] integrated meta-path selection with user-guidance to cluster objects in networks, where user provided seeds for each cluster acted as guidance. VEPathCluster [9] (Vertex/Edge-centric meta-Path Clustering) combined meta-path vertex-centric clustering with meta-path edge-centric clustering.

In addition, for some specific applications, researchers have integrated the topological structure of networks with graph clustering methods. A multivariate weighted complex network method [10] was applied in order to characterise the patterns in gas-liquid two-phase flow. A visibility graph model [11, 12] was designed for clustering multi-scale networks, and achieved a satisfactory clustering result when applied to detecting epileptic seizures from the EEG dataset and typical patterns form an oil-water two-phase flow dataset. For clustering heterogeneous information networks with incomplete attributes, a probabilistic clustering method [13] and a structural-based similarity measurement, namely NetSim [14], were developed.

Most existing methods have achieved good clustering results for the heterogeneous information networks with a specified simple network schema, but are ineffective in dealing with heterogeneous information networks with a general network schema or lacking network schema information. For example, in Fig 1B, Douban Movie network (a well-known movie recommender system in China http://movie.douban.com/) follows a general network schema, which contains six different types of objects: user, group, movie, actor, director and type, and the different relations between them. For such a heterogeneous information network with a general network schema, RankClus and NetClus are ineffective. In addition, the meta-path is difficult to choose for users. Another limitation of most existing methods is that they can only cluster one type of object at a time in the network. In other words, we must repeatedly apply the existing method to obtain the clustering results for different types of objects.

Recently, the theory of tensor factorization provided a new perspective of clustering analysis. A tensor is the general expression of a matrix, in which the elements are addressed by more than two indices. Tensor factorization based clustering has been used in computer graphics [15] and computer vision applications [16–21]. By bridging tensor factorization and clustering, we can obtain a fascinating methodology for mining heterogeneous information networks.

However, many heterogeneous information networks are very sparse, where most elements in the tensor are zeros. For example, in the DBLP database (Aug. 2015 version), there are 3,067,295 papers and 1,603,605 authors, but only 8,128,282 author-paper relations. That is to say, there are only 0.00017% nonzero elements in the huge sparse adjacent matrix of author and paper.

Another challenge is the curse of dimensionality [22]. It has been proven [23] that the distances or similarities between pairs of elements in the high dimensional tensor are almost the same for the vast majority of data distributions and distance functions. Therefore, most existing clustering methods cannot be used in the sparse and high dimensional heterogeneous information networks directly.

To solve the problem of clustering heterogeneous information networks with general network schemas or even without network schema information, e.g., Douban Movie network in Fig 1(b), and clustering all types of objects simultaneously in a single pass, we propose a sparse tensor factorization based method, which is called STFClus (**S**parse **T**ensor **F**actorization based **Clus**tering). We model a heterogeneous information network as a multi-way array, i.e., tensor. Each object type maps onto one mode of the tensor, and the relations between different types of objects map onto the elements in tensor. The main contributions made by our paper are as follows:

1. We propose a novel clustering framework based on sparse tensor factorization, namely STFClus, which can cluster heterogeneous information networks with general network schemas or even without network schema information. Another advantage is that STFClus can cluster all types of objects simultaneously in a single pass.
2. The clustering issue based on tensor factorization is modeled as an optimization problem, which is similar to the well-known Tucker decomposition [24, 25]. We propose an Alternating Least Squares (ALS) [26] algorithm to solve the clustering problem.
3. In STFClus, only nonzero tensor elements together with corresponding tensor indices are handled, and a non-distance function for similarity measurement between pairs of objects is needed.
4. We discuss the bottleneck of implementation for STFClus, and propose a performance improvement method that avoids the need to calculate large scale intermediate results. We also propose a feasible initialization method to start STFClus.
5. STFClus is tested on both synthetic and real-world networks. Experimental results show that STFClus outperforms the state-of-the-art baselines in terms of key performance indicators such as accuracy and efficiency.

## Methods

### Preliminaries

First, we introduce some related concepts and tensor notation that will be used in this paper. More details about tensor algebra can be found in [27–29].

A tensor is a multi-dimensional array. The order of a tensor is the number of dimensions, also known as ways or modes. We will follow the convention used in [27] to denote scalars by lowercase letters, e.g., *a*, *b*, *c*, vectors (one mode) by boldface lowercase letters, e.g., **a**, **b**, **c**, matrices (two modes) by boldface capital letters, e.g., **A**, **B**, **C**, and tensors (three modes or more) by boldface calligraphic letters, e.g., X,Y,Z. The **a**_*r*:_ denotes the *r*th row of matrix **A**, and **a**_:*r*_ denotes the *r*th column of matrix **A**. Elements of a matrix or a tensor are denoted by lowercase letters with subscripts, i.e., the (*i*_1_, *i*_2_, ⋯, *i*_*N*_)th element of an *N*th order tensor X is denoted by *x*_*i*_1_, *i*_2_, ⋯, *i*_*N*__.

Some common definitions for tensors are set out below, as used in [28].

***Definition 1*** (*Matricization*) [28]. Matricization transforms an *N*-order tensor into a matrix by arranging the elements in a particular order.

For example, the matricization of a tensor $X\in R^{I_{1}\times I_{2}\times \cdots \times I_{N}}$ along the *n*th mode is denoted as $X_{\left(n\right)}\in R^{I_{n}\times \left(I_{1}\times \cdots \times I_{n-1}\times I_{n+1}\times \cdots \times I_{N}\right)}$. A special case of matricization is vectorization, which transforms a tensor into a vector, i.e., all modes of the tensor become row modes. The vectorization of a tensor $X\in R^{I_{1}\times I_{2}\times \cdots \times I_{N}}$ is denoted by $\vec{X}\equiv X_{\left(\emptyset \right)}\in R^{\prod_{n=1}^{N}I_{n}}$.

***Definition 2*** (*Hadamard product*) [28]. The Hadamard product for two tensors with the same dimensions is also known as the element-wise product. For $X,Y\in R^{I_{1}\times I_{2}\times \cdots \times I_{N}}$, their Hadamard product is denoted by $X*Y\in R^{I_{1}\times I_{2}\times \cdots \times I_{N}}$, and its elements are given by ${\left(X*Y\right)}_{i_{1}i_{2}\cdots i_{N}}=x_{i_{1}i_{2}\cdots i_{N}}y_{i_{1}i_{2}\cdots i_{N}}$.

***Definition 3*** (*Kronecker product*) [28]. The Kronecker product for two matrices $A\in R^{I\times J}$ and $B\in R^{K\times L}$ is denoted by **A** ⊗ **B**, which is a matrix of size (*IJ*) × (*KL*) and defined by

$$\begin{array}{c} A⊗B=\left[\begin{array}{cccc} a_{11}B & a_{12}B & \cdots & a_{1J}B\\ a_{21}B & a_{22}B & \cdots & a_{2J}B\\ \vdots & \vdots & \ddots & \vdots \\ a_{I1}B & a_{I2}B & \cdots & a_{IJ}B \end{array}\right] \end{array}$$

***Definition 4*** (*Inner product*) [28]. The inner product for two tensors with the same dimension, $X,Y\in R^{I_{1}\times I_{2}\times \cdots \times I_{N}}$, is denoted by 〈X,Y〉. The result of the inner product is the sum of all elements in their Hadamard product, and defined as

$$\begin{array}{c} \left\langle X,Y\right\rangle =\sum_{i_{1}=1}^{I_{1}}\sum_{i_{2}=1}^{I_{2}}\cdots \sum_{i_{N}=1}^{I_{N}}{\left(X*Y\right)}_{i_{1}i_{2}\cdots i_{N}} \end{array}$$

***Definition 5*** (*Frobenius norm*) [28]. The Frobenius norm for a tensor $X\in R^{I_{1}\times I_{2}\times \cdots \times I_{N}}$ is defined as ${∥X∥}_{F}=\sqrt{\langle X,X\rangle }$.

***Definition 6*** (*Mode-n matrix product*) [28]. The Mode-n matrix product of a tensor $X\in R^{I_{1}\times I_{2}\times \cdots \times I_{N}}$ with a matrix $U\in R^{J\times I_{n}}$ is denoted by $X\times_{n}U$ and is of size *I*_1_ × ⋯ × *I*_*n*−1_ × *J* × *I*_*n*+1_ × ⋯ × *I*_*N*_. Its elements are given by ${\left(X\times_{n}U\right)}_{i_{1}\cdots i_{n-1}ji_{n+1}\cdots i_{N}}=\sum_{i_{n}=1}^{I_{n}}x_{i_{1}i_{2}\cdots i_{N}}u_{ji_{n}}$.

The Mode-n matrix product of a tensor $X\in R^{I_{1}\times I_{2}\times \cdots \times I_{N}}$ with a matrix $U\in R^{J\times I_{n}}$ is equivalent to first matricization of X along the *n*th mode, followed by the matrix multiplication of $X_{\left(n\right)}$ with **U**, before finally folding the result back as a tensor.

Given an *N*th order tensor $X\in R^{I_{1}\times I_{2}\times \cdots \times I_{N}}$, the Tucker decomposition [24] of X yields a core tensor G of specified size *J*_1_ × *J*_2_ × ⋯ × *J*_*n*_, *J*_*n*_ ≤ *I*_*n*_ and factor matrices $U^{\left(n\right)}\in R^{I_{n}\times J_{n}},n=1,2,\cdots ,N$, such that

$$\begin{array}{c} G\times_{1}U^{\left(1\right)}\times_{2}U^{\left(2\right)}\times_{3}\cdots \times_{N}U^{\left(N\right)}\equiv \left[\;\left[G;U^{\left(1\right)},U^{\left(2\right)},\cdots ,U^{\left(N\right)}\right]\;\right] \end{array}$$

The Tucker decomposition approximates a tensor as a series of Mode-n matrix products of a smaller core tensor with a factor matrix along each mode. In traditional Tucker decomposition, the factor matrices ${\left\{U^{\left(n\right)}\right\}}_{n=1}^{N}$ are assumed to be orthogonal.

We now give the definition for an information network, which is based on work by Y. Sun and J. Han [3, 5].

***Definition 7*** (*Information network*) [3]. An **information network** is a weighted graph defined on a set of objects belonging to *T* types, denoted by $V={\left\{V_{t}\right\}}_{t=1}^{T}$, a set of binary relations on V, denoted by *E*, and a weight mapping function, denoted by $W:E\to R^{+}$. The information network is denoted by G=(V,E,W). Specially, when *T* ≥ 2, the information network is called as **heterogeneous information network**, otherwise, it is called as **homogeneous information network**.

We denote each object of type $V_{t}$ as ${\left\{\upsilon_{n}^{t}\right\}}_{n=1}^{N_{t}}$, where *N*_*t*_ is the number of objects in type $V_{t}$, i.e., $N_{t}=\left|V_{t}\right|$ and *t* = 1, 2, ⋯, *T*. The total number of objects in the network is given by $N=\sum_{t=1}^{T}N_{t}$. For an arbitrary edge $e_{i,j}^{\left(t_{a},t_{b}\right)}=\langle \upsilon_{i}^{t_{a}},\upsilon_{j}^{t_{b}}\rangle \in E,t_{a}\neq t_{b}$, the simplest weight mapping function $W:E\to R^{+}$ can be defined as follows:

$$\begin{array}{c} \omega_{i,j}^{\left(t_{a},t_{b}\right)}=W\left(e_{i,j}^{\left(t_{a},t_{b}\right)}\right)=1 \end{array}$$ (1)

**Fig 2:**
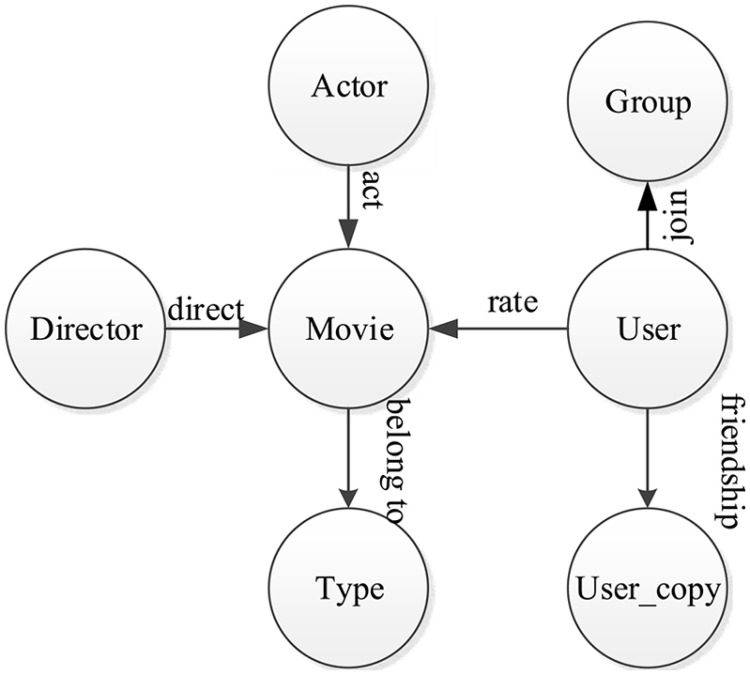
The revised network schema of a Douban Movie network. We take a copy of users, denoted as user_copy, and let the friendship relations appear only between user and user_copy.

In particular, we need to give some restrictions for heterogeneous information networks in our work. Firstly, each edge $e_{i,j}^{\left(t_{a},t_{b}\right)}=\langle \upsilon_{i}^{t_{a}},\upsilon_{j}^{t_{b}}\rangle \in E$ only appears on different types of objects, i.e., *t*_*a*_ ≠ *t*_*b*_. Secondly, we assume that the heterogeneous information network G=(V,E,W) is undirected, i.e., $e_{i,j}^{\left(t_{a},t_{b}\right)}=e_{j,i}^{\left(t_{b},t_{a}\right)}$. It is noteworthy that many edges in real-world applications appear on objects of the same type. An example is the friendship relation type between users in a Douban Movie network, as shown in Fig 1(b). In this case, we can take a copy of this type of object, and let the edge appear only between the two types of objects. In the Douban Movie network, we can take a copy of users and denote it as user_copy. Then, we can let the friendship relations appear only between user and user_copy. The revised network schema of the Douban Movie network is shown in Fig 2. In the following sections, the heterogeneous information network G=(V,E,W) will comply with these restrictions, unless there are special instructions.

### Sparse tensor factorization based clustering

#### Tensor construction and sparse representation

The relationships in heterogeneous information networks show a semantic link in real-world applications, which is defined as follows:

***Definition 8*** (*Relationship in Heterogeneous Information Network*). Given a heterogeneous information network G=(V,E,W), a relationship *R* is a connected sub-graph of *G*, denoted by R=(V′,E′,W′), where $V^{\prime }=\{\upsilon_{n_{1}}^{1},\upsilon_{n_{2}}^{2},\cdots ,\upsilon_{n_{T}}^{T}\}$, 0 ≤ *n*_*t*_ ≤ *N*_*t*_, *t* = 1, 2, ⋯, *T*, *E*′ ⊆ *E* is a binary relation on V′, and *W*′ = *W*.

For example, a semantic relation in a real-world bibliographic network (in Fig 1A, containing four types of objects {*A*, *P*, *V*, *T*}), “an Author $\upsilon_{i}^{A}$ writes a Paper $\upsilon_{j}^{P}$ published in the Venue $\upsilon_{m}^{V}$, and containing the Term $\upsilon_{n}^{T}$”, can be represented by a relationship $R=(\{\upsilon_{i}^{A},\upsilon_{j}^{P},\upsilon_{m}^{V},\upsilon_{n}^{T}\},\{\langle \upsilon_{i}^{A},\upsilon_{j}^{P}\rangle ,\langle \upsilon_{j}^{P},\upsilon_{m}^{V}\rangle ,\langle \upsilon_{j}^{P},\upsilon_{n}^{T}\rangle \},W)$. we can use the subscript of each object in *R* to mark the corresponding relationship. In this example, the relationship can be marked by *R*_*i*, *j*, *m*, *n*_.

**Fig 3:**
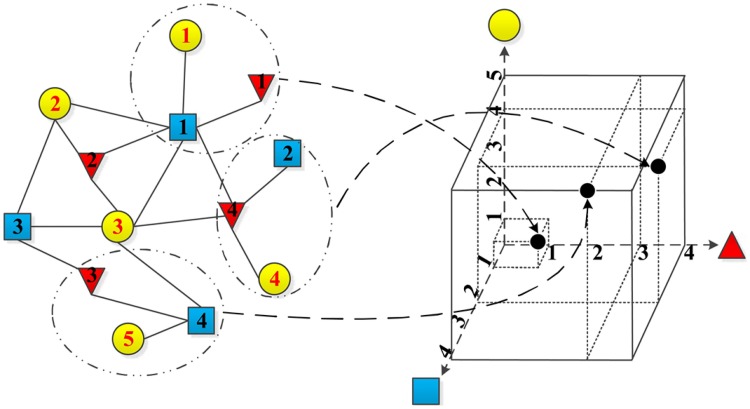
An example of tensor construction from a given heterogeneous information network. On the left is the original network with three types of objects (yellow circle, blue square and red triangle), and on the right cube is the constructed 3-order tensor. The number within each object is the object identifier. Each element (black dot in the right cube) in the tensor represents a relationship in the network (black dashed circle in the left).

Let X be a *T*th order tensor of size *N*_1_ × *N*_2_ × ⋯ × *N*_*T*_, each mode of X representing one type of object in the network *G*. An arbitrary element, *x*_*n*_1_*n*_2_⋯*n*_*T*__ ≥ 0, for *n*_*t*_ = 1, 2, ⋯, *N*_*t*_, is the weight of the corresponding relationship *R*_*n*_1_, *n*_2_, ⋯, *n*_*T*__ that exists, i.e.,

$$x_{n_{1}\;n_{2}\cdots n_{T}}=\{\begin{array}{ll} \underset{e_{i,j}\in E\prime }{⊠}\omega_{i,j} & \text{if }\exists R_{n_{1},n_{2},\cdots ,n_{T}};\\ 0 & \text{otherwise}. \end{array}$$ (2)

where ⊠ is an operation on the weights of all edges in *R*_*n*_1_, *n*_2_, ⋯, *n*_*T*__. In the simplest example, ⊠ can be defined as $\underset{e_{i,j}\in E^{\prime }}{⊠}\omega_{i,j}=1$. The heterogeneous information network G=(V,E,W) can then be represented in tensor form as X. The method of determining whether the relationship *R*_*n*_1_, *n*_2_, ⋯, *n*_*T*__ actually exists is related to graph theory and will not be discussed here. Fig 3 gives an example of tensor construction.

To deal with the sparse tensor X, we use the coordinate format as proposed in [30]. Assuming there are *J* non-zero elements in X, then a vector $z\in R^{J}$ and a matrix $M\in R^{J\times T}$ can represent the value and the corresponding coordinates of each non-zero element in X respectively. Here the *j*th non-zero value is given by *z*_*j*_ and its subscript is given by the *j*th row of **M**, i.e., **m**_*j*:_. Let *x*_*n*_1_*n*_2_⋯*n*_*T*__ be the *j*th non-zero element in X, we have **m**_*j*:_ = [*m*_*j*_1__, *m*_*j*_2__, ⋯, *m*_*j*_*T*__] = [*n*_1_, *n*_2_, ⋯, *n*_*T*_] and *z*_*j*_ = *x*_*n*_1_*n*_2_⋯*n*_*T*__. In other words, *m*_*j*_*t*__ = *n*_*t*_ represents the fact that the *t*th coordinate of the *j*th non-zero element in X is *n*_*t*_, and that the value of the *j*th non-zero element in X is *z*_*j*_. This sparse representation of tensors is the same as the format implemented in the MATLAB Tensor Toolbox [31].

#### Problem formulation

Given a heterogeneous information network G=(V,E,W), the tensor representation X is usually large and sparse. We can use the sparse representation for X, i.e., the *J* non-zero weight elements vector $z\in R^{J}$ and the corresponding coordinates matrix $M\in R^{J\times T}$. Each row of **M** can be treated as a relationship in the network, and the corresponding element in **z** is the weight of the relationship.

So all the rows of the coordinates matrix $M={\left[m_{1:}^{⊤},m_{2:}^{⊤},\cdots ,m_{J:}^{⊤}\right]}^{⊤}$, **m**_*j*:_ = [*m*_*j*_1__, *m*_*j*_2__, ⋯, *m*_*j*_*T*__], for *j* = 1, 2, ⋯, *J*, represent the input relationships in the network, which we want to partition into *K* sub-tensors (clusters) $\left\{C_{1},C_{2},\cdots ,C_{K}\right\}$. The vector **z** = [*z*_1_, *z*_2_, ⋯, *z*_*J*_] is the weight vector for the input relationships. The centre of the cluster $C_{k}$ is denoted by **c**_*k*_ = [*c*_*k*_1__, *c*_*k*_2__, ⋯, *c*_*k*_*T*__], for *k* = 1, 2, ⋯, *K*. Let *y*_*i*_ ∈ {1, 2, ⋯, *K*} be the associated unknown cluster label. For example *y*_*j*_ = *k* represents **m**_*j*:_ belonging to the *k*th cluster, and *y*_*j*_*t*__ = *k*′ represents the subscript *m*_*j*_*t*__ of **m**_*j*:_ (that is the *j*_*t*_th object of type $V_{t}$ in *G*) belonging to the *k*′th cluster.

Generally speaking, a relationship (or a sub-graph) in the heterogeneous information network may belong to several clusters. Meanwhile, the objects in the relationship may also belong to more than one cluster. We assume that there is already a way to measure the probability that the objects or relationships belong to a specific cluster. Let’s denote *p*_*j*_*t*_, *k*_ = *P*(*y*_*j*_*t*__ = *k*|*m*_*j*_*t*__) as the probability that the *t*th component of the point **m**_*j*:_ belongs to the *k*th cluster, and *p*_*j*, *k*_ = *P*(*y*_*j*_ = *k*|**m**_*j*:_) as the probability that the point **m**_*j*:_ belongs to the *k*th cluster.

A basic clustering approach minimizes the sum of differences between individual relationships in each cluster and the corresponding cluster centres. So the heterogeneous information network clustering problem can be formalized by the vectorized version as follows:

$$\begin{array}{c} \underset{p_{j,k}}{\text{min}}{\sum_{j=1}^{J}\left\|z_{j}m_{j:}-z_{j}\sum_{k=1}^{K}p_{j,k}c_{k}\right\|}_{F}^{2}=\underset{p_{j,k}}{\text{min}}\sum_{j=1}^{J}z_{j}^{2}{\left\|m_{j:}-\sum_{k=1}^{K}p_{j,k}c_{k}\right\|}_{F}^{2}\\ s.t.\;\{\begin{array}{c} \begin{array}{l} \forall j,\sum_{k=1}^{K}p_{j,k}=1\\ \forall j,\forall k,p_{j,k}\in \left[0,1\right] \end{array} \end{array} \end{array}$$ (3)

In Eq (3), $z_{j}^{2}>0$ and ${\left\|m_{j:}-\sum_{k=1}^{K}p_{j,k}c_{k}\right\|}_{F}^{2}\geq 0$, so $\underset{p_{j,k}}{\text{min}}\sum_{j=1}^{J}z_{j}^{2}{\left\|m_{j:}-\sum_{k=1}^{K}p_{j,k}c_{k}\right\|}_{F}^{2}=\sum_{j=1}^{J}\underset{p_{j,k}}{\text{min}}\left(z_{j}^{2}{\left\|m_{j:}-\sum_{k=1}^{K}p_{j,k}c_{k}\right\|}_{F}^{2}\right)$. Since $z_{j}^{2}>0$, the optimal solution $p_{j,k}^{*}$ of $\underset{p_{j,k}}{\text{min}}\left(z_{j}^{2}{\left\|m_{j:}-\sum_{k=1}^{K}p_{j,k}c_{k}\right\|}_{F}^{2}\right)$ is also the optimal solution for $\underset{p_{j,k}}{\text{min}}\left({\left\|m_{j:}-\sum_{k=1}^{K}p_{j,k}c_{k}\right\|}_{F}^{2}\right)$. In other words, $\text{arg}\underset{p_{j,k}}{\text{min}}\left(z_{j}^{2}{\left\|m_{j:}-\sum_{k=1}^{K}p_{j,k}c_{k}\right\|}_{F}^{2}\right)=\text{arg}\underset{p_{j,k}}{\text{min}}\left({\left\|m_{j:}-\sum_{k=1}^{K}p_{j,k}c_{k}\right\|}_{F}^{2}\right)$. So we can ignore $z_{j}^{2}$ in Eq (3). Also we can re-write Eq (3) by a new perspective of sparse form as follows:

$$\begin{array}{l} \underset{p_{j_{t},k}}{\text{min}}{\sum_{j=1}^{J}\sum_{t=1}^{T}\left\|m_{j_{t}}-\sum_{k=1}^{K}p_{j_{t},k}c_{k_{t}}\right\|}_{F}^{2}\\ s.t.\;\{\begin{array}{c} \begin{array}{l} \forall t,\forall j,\sum_{k=1}^{K}p_{j_{t},k}=1\\ \forall t,\forall j,\forall k,p_{j_{t},k}\in \left[0,1\right] \end{array} \end{array} \end{array}$$ (4)

Actually, Eq (3) aims to cluster relationships in heterogeneous information networks, and Eq (4) partitions different types of objects into *K* clusters.

Now we form *T* matrices, denoted by $U^{\left(t\right)}\in R^{N_{t}\times K}$, for *t* = 1, 2, ⋯, *T*. The element $u_{i,k}^{\left(t\right)}\in U^{\left(t\right)}$, for *i* = 1, 2, ⋯, *N*_*t*_; *t* = 1, 2, ⋯, *T*; *k* = 1, 2, ⋯, *K*, can be defined as $u_{i,k}^{\left(t\right)}=p_{j_{t},k}$, if *i* = *j*_*t*_; otherwise, $u_{i,k}^{\left(t\right)}=0$. Then, $u_{i,k}^{\left(t\right)}$ represents the probability that the *i*th object in type $V_{t}$, i.e., $\upsilon_{i}^{t}$, belongs to the *k*th cluster. So the matrix $U^{\left(t\right)}\in R^{N_{t}\times K}$ is the projection matrix for the corresponding mode of X. Then a new small size tensor $G\in R^{\overset{T}{\overset{︷}{K\times K\times \cdots \times K}}}$ is used as the mixture coefficient among different modes and clusters.

Let G be the core tensor and **U**^(*t*)^, *t* = 1, 2, ⋯, *T* be the factor matrices, we can use $\left[\;\left[G;U^{\left(1\right)},U^{\left(2\right)},\cdots ,U^{\left(T\right)}\right]\;\right]$ to approximate X, i.e., $X\approx [\;\left[G;U^{\left(1\right)},U^{\left(2\right)},\cdots ,U^{\left(T\right)}\right]\;]$. Then, we can formalize the clustering problem in a way that is similar to the Tucker decomposition in [32].

$$\begin{array}{c} \underset{G,U^{\left(1\right)},U^{\left(2\right)},\cdots ,U^{\left(T\right)}}{\text{min}}{\left\|X-〚G;U^{\left(1\right)},U^{\left(2\right)},\cdots ,U^{\left(T\right)}〛\right\|}_{F}^{2}\\ s.t.\;\{\begin{array}{c} \begin{array}{l} \forall t,\sum_{k=1}^{K}u_{ik}^{\left(t\right)}=1\\ \forall t,\forall i,\forall k,u_{ik}^{\left(t\right)}\in \left[0,1\right]\\ \forall t,rank\left(U^{\left(t\right)}\right)=K \end{array} \end{array} \end{array}$$ (5)

In Eq (5), *i* = 1, 2, ⋯, *N*_*t*_;*t* = 1, 2, ⋯, *T*;*k* = 1, 2, ⋯, *K*, and *K* < min{*N*_1_, *N*_2_, ⋯, *N*_*T*_} is the total number of clusters. The first constraint in Eq (5) guarantees that the sum of probabilities for each object belonging to all clusters is 1. The second constraint in Eq (5) stipulates that each probability should be in the range [0, 1]. The last constraint in Eq (5) ensures that each factor matrix is of full column rank, i.e., for any mode, there is no empty cluster and any two clusters are not the same.

In fact, Eq (5) can achieve the results of Eqs (3) and (4) simultaneously. That is, Eq (5) clusters different types of objects and relationships in a heterogeneous information network simultaneously. The factor matrices **U**^(1)^,**U**^(2)^, ⋯,**U**^(*T*)^ are the cluster indication matrices for the *T* types of objects respectively and the probability of relationship *R*_*n*_1_, *n*_2_, ⋯, *n*_*T*__ belonging to the *k*th cluster is given by $g_{k,k,\cdots ,k}u_{n_{1},k}^{\left(1\right)}u_{n_{2},k}^{\left(2\right)}\cdots u_{n_{T},k}^{\left(T\right)}$, where $g_{k,k,\cdots ,k}\in G$ and $u_{n_{t},k}^{\left(t\right)}\in U^{\left(t\right)}$.

#### Algorithm for STFClus

The Alternating Least Squares (ALS) method is a common approach for solving the Tucker decomposition problem. It updates one factor matrix iteratively at each round, while keeping the other factor matrices unchanged. The proposed algorithm for STFClus is also an ALS method that consists of two stages: the factor matrices updating and the core tensor updating. After the core tensor and all factor matrices are initialized, all variables in Eq (5) are fixed in the factor matrices updating stage, except for the mode-*t* factor matrix **U**^(*t*)^. Then an approach similar to NMF is applied to search for the optimal **U**^(*t*)^ that minimizes the objective function. In the core tensor updating stage, using the optimal factor matrices obtained by the factor matrices updating stage, the core tensor is updated. Finally, the factor matrices updating and core tensor updating stages are iteratively implemented until the approximation error in the objective function is unchanged. The details of the tensor algebra and properties used in the algorithm can be found in [29].

In the factor matrices updating stage, each mode-*t* factor matrix **U**^(*t*)^ is obtained, while the core tensor and other factor matrices are fixed. The objective function in Eq (5) can be rewritten by matricization of X along the *t*th mode as follows:

$$\begin{array}{c} \underset{U^{\left(t\right)}}{\text{min}}{∥X_{\left(t\right)}-U^{\left(t\right)}{\left[\;\left[G;U^{\left(1\right)},\cdots ,U^{\left(t-1\right)},U^{\left(t+1\right)},\cdots ,U^{\left(T\right)}\right]\;\right]}_{\left(t\right)}∥}_{F}^{2} \end{array}$$ (6)

where $X_{\left(t\right)}\in R^{N_{t}\times \left(N_{1}\times \cdots \times N_{t-1}\times N_{t+1}\times \cdots \times N_{T}\right)}$.

If we assume that the optimal solution **U**^(*t*)^ satisfies all the constraints in Eq (5), then Eq (6) can be written as the following linear equation:

$$\begin{array}{cc} X_{\left(t\right)} & =U^{\left(t\right)}{〚G;U^{\left(1\right)},\cdots ,U^{\left(t-1\right)},U^{\left(t+1\right)},\cdots ,U^{\left(T\right)}〛}_{\left(t\right)}\\ & =U^{\left(t\right)}G_{\left(t\right)}{\left(U^{\left(T\right)}⊗\cdots ⊗U^{\left(t+1\right)}⊗U^{\left(t-1\right)}⊗\cdots ⊗U^{\left(1\right)}\right)}^{⊤} \end{array}$$ (7)

We denote S as the tensor $\left[\;\left[G;U^{\left(1\right)},\cdots ,U^{\left(t-1\right)},U^{\left(t+1\right)},\cdots ,U^{\left(T\right)}\right]\;\right]$. Then, $S\in R^{N_{1}\times \cdots \times N_{t-1}\times K\times N_{t+1}\times \cdots \times N_{T}}$, and the matricization of S along the *t*th mode is

$$\begin{array}{c} S_{\left(t\right)}=G_{\left(t\right)}{\left(U^{\left(T\right)}⊗\cdots ⊗U^{\left(t+1\right)}⊗U^{\left(t-1\right)}⊗\cdots ⊗U^{\left(1\right)}\right)}^{⊤} \end{array}$$ (8)

where $S_{\left(t\right)}\in R^{K\times (N_{1}\times \cdots \times N_{t-1}\times N_{t+1}\times \cdots \times N_{T})}$. Now Eq (7) is similar to the NMF problem in [33, 34], i.e.,

$$\begin{array}{c} X_{\left(t\right)}=U^{\left(t\right)}S_{\left(t\right)} \end{array}$$ (9)

Thus, we can use the NMF update rule in [34] to update **U**^(*t*)^ as follows:

$$\begin{array}{c} U^{\left(t\right)}\leftarrow U^{\left(t\right)}*\frac{X_{\left(t\right)}S_{\left(t\right)}^{⊤}}{U^{\left(t\right)}S_{\left(t\right)}S_{\left(t\right)}^{⊤}} \end{array}$$ (10)

where the symbol $\frac{\left(•\right)}{\left(•\right)}$ denotes the element-wise division of two matrices with the same size. Note that the factor matrices derived by Eq (10) do not satisfy the first and second constraints in Eq (5). To satisfy these two constraints, we can normalize each row of the factor matrices.

$$\begin{array}{c} u_{i,k}^{\left(t\right)}\leftarrow \frac{u_{i,k}^{\left(t\right)}}{\sum_{k=1}^{K}u_{i,k}^{\left(t\right)}} \end{array}$$ (11)

In the core tensor updating stage, we keep all the factor matrices unchanged and rewrite the objective function in Eq (5) by vectorization of X as follows:

$$\begin{array}{c} \underset{G}{\text{min}}{\left\|X-〚G;U^{\left(1\right)},U^{\left(2\right)},\cdots ,U^{\left(T\right)}〛\right\|}_{F}^{2}=\underset{\vec{G}}{\text{min}}{\left\|\vec{X}-\left(U^{\left(T\right)}⊗\cdots ⊗U^{\left(1\right)}\right)\vec{G}\right\|}_{F}^{2} \end{array}$$ (12)

We assume that all the factor matrices satisfy the constraints in Eq (5). Then the core tensor G in Eq (12) can be obtained by solving the following linear equation:

$$\begin{array}{c} \vec{X}=\left(U^{\left(T\right)}⊗\cdots ⊗U^{\left(1\right)}\right)\vec{G} \end{array}$$ (13)

We set:

$$\begin{array}{c} Q=U^{\left(T\right)}⊗\cdots ⊗U^{\left(1\right)} \end{array}$$ (14)

where $Q\in R^{\left(\prod_{t=1}^{T}N_{t}\right)\times K^{T}}$. Then, we can transform Eq (13) to a NMF model, i.e.,

$$\begin{array}{c} \vec{X}=Q\vec{G} \end{array}$$ (15)

Thus, the NMF update rule in [34] can be used to update $\vec{G}$ as follows:

$$\begin{array}{c} \vec{G}\leftarrow \vec{G}*\frac{Q^{⊤}\vec{X}}{Q^{⊤}Q\vec{G}} \end{array}$$ (16)

where

$$\begin{array}{lll} Q^{⊤}\vec{X} & = & \vec{X{\left(Q^{⊤}\right)}^{⊤}}\\ & = & \vec{X{\left({\left(U^{\left(T\right)}\right)}^{⊤}⊗\cdots ⊗{\left(U^{\left(1\right)}\right)}^{⊤}\right)}^{⊤}}\\ & = & \vec{〚X;{\left(U^{\left(1\right)}\right)}^{⊤},\cdots ,{\left(U^{\left(T\right)}\right)}^{⊤}〛} \end{array}$$ (17)

and

$$\begin{array}{lll} Q^{⊤}Q\vec{G} & = & {\left(U^{\left(T\right)}⊗\cdots ⊗U^{\left(1\right)}\right)}^{⊤}\left(U^{\left(T\right)}⊗\cdots ⊗U^{\left(1\right)}\right)\vec{G}\\ & = & \left({\left(U^{\left(T\right)}\right)}^{⊤}U^{\left(T\right)}⊗\cdots ⊗{\left(U^{\left(1\right)}\right)}^{⊤}U^{\left(1\right)}\right)\vec{G}\\ & = & \vec{〚G;{\left(U^{\left(1\right)}\right)}^{⊤}U^{\left(1\right)},\cdots ,{\left(U^{\left(T\right)}\right)}^{⊤}U^{\left(T\right)}〛} \end{array}$$ (18)

The properties of Kronecker products and vectorization operators can be found in [35]. Then, Eq (16) is equal to:

$$\begin{array}{lll} \vec{G} & \leftarrow & \vec{G}*\frac{\vec{〚X;{\left(U^{\left(1\right)}\right)}^{⊤},\cdots ,{\left(U^{\left(T\right)}\right)}^{⊤}〛}}{\vec{〚G;{\left(U^{\left(1\right)}\right)}^{⊤}U^{\left(1\right)},\cdots ,{\left(U^{\left(T\right)}\right)}^{⊤}U^{\left(T\right)}〛}}\\ & = & \vec{G*\frac{〚X;{\left(U^{\left(1\right)}\right)}^{⊤},\cdots ,{\left(U^{\left(T\right)}\right)}^{⊤}〛}{〚G;{\left(U^{\left(1\right)}\right)}^{⊤}U^{\left(1\right)},\cdots ,{\left(U^{\left(T\right)}\right)}^{⊤}U^{\left(T\right)}〛}} \end{array}$$ (19)

According to Eq (19), we can get the update rule of the core tensor G as follows:

$$\begin{array}{c} G\leftarrow G*\frac{\left[\;\left[X;{\left(U^{\left(1\right)}\right)}^{⊤},\cdots ,{\left(U^{\left(T\right)}\right)}^{⊤}\right]\;\right]}{\left[\;\left[G;{\left(U^{\left(1\right)}\right)}^{⊤}U^{\left(1\right)},\cdots ,{\left(U^{\left(T\right)}\right)}^{⊤}U^{\left(T\right)}\right]\;\right]} \end{array}$$ (20)

#### Feasibility and convergence analysis

First, we discuss the feasibility of STFClus.

***Theorem 1***: The STFClus optimization problem is equivalent to the optimization problem in Eq (4).

Before giving the proof of Theorem 1, we first review the clustering problem as defined in Eq (4). Eq (4) is a sparse form, which partitions each object into different clusters. The *p*_*j*_*t*_, *k*_ is the cluster indicator, which gives the probability of an object belonging to the corresponding cluster. In the matrix form, clustering *t*th type of objects can be formalized as:

$$\begin{array}{c} \underset{P}{\text{min}}{∥M-PC∥}_{F}^{2} \end{array}$$

where **P** is the cluster indication matrix for the *t*th type of objects, and **C** is the cluster centres.

*Proof*. Since Eq (6) is transformed from Eq (5) for updating **U**^(*t*)^, Eq (5) can be rewritten as:

$$\begin{array}{c} \underset{U^{\left(t\right)}}{\text{min}}{∥X_{\left(t\right)}-U^{\left(t\right)}S_{\left(t\right)}∥}_{F}^{2} \end{array}$$

where **U**^(*t*)^ is the cluster indication matrix for the *t*th type of objects, and $S_{\left(t\right)}$ is the cluster centres.

$X_{\left(t\right)}$ is the matricization of X along the *t*th mode, and **M** is the sparse representation of X, and let **P** = **U**^(*t*)^, $C=S_{\left(t\right)}$, so Eq (5) will have the same form as Eq (4).

Then, we give the convergence analysis of STFClus. Since Lee and Seung have proven the convergence of NMF in [34], we cite theorem 1 in [34] as **Theorem 2** in this paper.

***Theorem 2*** [34]: The function $∥X_{\left(t\right)}-U^{\left(t\right)}S_{\left(t\right)}{∥}_{F}^{2}$ is non-increasing under the update rule $U^{\left(t\right)}\leftarrow U^{\left(t\right)}*\frac{X_{\left(t\right)}S_{\left(t\right)}^{⊤}}{U^{\left(t\right)}S_{\left(t\right)}S_{\left(t\right)}^{⊤}}$. And the function $∥X_{\left(t\right)}-U^{\left(t\right)}S_{\left(t\right)}{∥}_{F}^{2}$ is invariant if and only if **U**^(*t*)^ is at a local minima.

*Proof*. See the details in [34].

By extending **Theorem 2** to high-dimensional space, we prove that STFClus is stable.

***Lemma 1***: The objective function ${\left\|X-〚G;U^{\left(1\right)},U^{\left(2\right)},\cdots ,U^{\left(T\right)}〛\right\|}_{F}^{2}$ in Eq (5) is non-increasing under the update rules $U^{\left(t\right)}\leftarrow U^{\left(t\right)}*\frac{X_{\left(t\right)}S_{\left(t\right)}^{⊤}}{U^{\left(t\right)}S_{\left(t\right)}S_{\left(t\right)}^{⊤}}$. And the function ${\left\|X-〚G;U^{\left(1\right)},U^{\left(2\right)},\cdots ,U^{\left(T\right)}〛\right\|}_{F}^{2}$ is invariant if and only if **U**^(*t*)^ is at a local minima.

*Proof*. We denote $U_{iter+1}^{\left(t\right)}$ and $U_{iter}^{\left(t\right)}$ as the solutions of the adjacent two iterations respectively, i.e., $U_{iter+1}^{\left(t\right)}=U_{iter}^{\left(t\right)}*\frac{X_{\left(t\right)}S_{\left(t\right)}^{⊤}}{U_{iter}^{\left(t\right)}S_{\left(t\right)}S_{\left(t\right)}^{⊤}}$. According to **Theorem 2**, we have

$$\begin{array}{c} {∥X_{\left(t\right)}-U_{iter+1}^{\left(t\right)}S_{\left(t\right)}∥}_{F}^{2}\leq {∥X_{\left(t\right)}-U_{iter}^{\left(t\right)}S_{\left(t\right)}∥}_{F}^{2}, \end{array}$$

where the equality holds if and only if $U_{iter+1}^{\left(t\right)}=U_{iter}^{\left(t\right)}$ and $U_{iter}^{\left(t\right)}$ is at a local minima. By substituting Eq (8) into this inequation, we obtain:

$$\begin{array}{cc} & {\left\|X_{\left(t\right)}-U_{iter+1}^{\left(t\right)}G_{\left(t\right)}{\left(U^{\left(T\right)}⊗\cdots ⊗U^{\left(t+1\right)}⊗U^{\left(t-1\right)}⊗\cdots ⊗U^{\left(1\right)}\right)}^{⊤}\right\|}_{F}^{2}\\ \leq & {\left\|X_{\left(t\right)}-U_{iter}^{\left(t\right)}G_{\left(t\right)}{\left(U^{\left(T\right)}⊗\cdots ⊗U^{\left(t+1\right)}⊗U^{\left(t-1\right)}⊗\cdots ⊗U^{\left(1\right)}\right)}^{⊤}\right\|}_{F}^{2} \end{array}$$

Then, fold the result back as a tensor:

$${\left\|X-〚G;U^{\left(1\right)},\cdots ,U_{iter+1}^{\left(t\right)},\cdots ,U^{\left(T\right)}〛\right\|}_{F}^{2}\begin{array}{c} \leq {\left\|X-〚G;U^{\left(1\right)},\cdots ,U_{iter}^{\left(t\right)},\cdots ,U^{\left(T\right)}〛\right\|}_{F}^{2}, \end{array}$$

where the equality holds if and only if $U_{iter+1}^{\left(t\right)}=U_{iter}^{\left(t\right)}$ and $U_{iter}^{\left(t\right)}$ is at a local minima.

By reversing the roles of **U**^(*t*)^ and G, the update rule of the core tensor in Eq (20) can be similarly proven.

### Implementation issues

#### Performance improvement

The bottleneck of the STFClus lies in the calculation of $S_{\left(t\right)}$. According to Eq (8), we need to compute the Kronecker products of *T* − 1 dense factor matrices. The intermediate results of the Kronecker products are dense and may be of very large size. The largest intermediate results of the Kronecker products would have $\underset{t}{\text{max}}\left(K^{T-1}\prod_{\underset{i\neq t}{i\in \left\{1,2,\cdots ,T\right\}}}N_{i}\right)$ elements, i.e., the time and space complexities are high.

In fact, the Kronecker products need not be calculated here. According to Eq (8), we can rewrite the $X_{\left(t\right)}S_{\left(t\right)}^{⊤}$ and $S_{\left(t\right)}S_{\left(t\right)}^{⊤}$ in Eq (10) as the following form.

$$\begin{array}{c} \begin{array}{cc} & X_{\left(t\right)}S_{\left(t\right)}^{⊤}\\ = & X_{\left(t\right)}{\left(G_{\left(t\right)}{\left(U^{\left(T\right)}⊗\cdots ⊗U^{\left(t+1\right)}⊗U^{\left(t-1\right)}⊗\cdots ⊗U^{\left(1\right)}\right)}^{⊤}\right)}^{⊤}\\ = & X_{\left(t\right)}{\left({\left(U^{\left(T\right)}\right)}^{⊤}⊗\cdots ⊗{\left(U^{\left(t+1\right)}\right)}^{⊤}⊗{\left(U^{\left(t-1\right)}\right)}^{⊤}⊗\cdots ⊗{\left(U^{\left(1\right)}\right)}^{⊤}\right)}^{⊤}{\left(G_{\left(t\right)}\right)}^{⊤}\\ = & {\left[\;\left[X;{\left(U^{\left(1\right)}\right)}^{⊤},\cdots ,{\left(U^{\left(t-1\right)}\right)}^{⊤},{\left(U^{\left(t+1\right)}\right)}^{⊤},\cdots ,{\left(U^{\left(T\right)}\right)}^{⊤}\right]\;\right]}_{\left(t\right)}{\left(G_{\left(t\right)}\right)}^{⊤} \end{array} \end{array}$$ (21)

$$\begin{array}{c} \begin{array}{cc} & S_{\left(t\right)}S_{\left(t\right)}^{⊤}\\ = & G_{\left(t\right)}{\left(U^{\left(T\right)}⊗\cdots ⊗U^{\left(t+1\right)}U^{\left(t-1\right)}⊗\cdots ⊗U^{\left(1\right)}\right)}^{⊤}{\left({\left(U^{\left(T\right)}\right)}^{⊤}⊗\cdots ⊗{\left(U^{\left(t+1\right)}\right)}^{⊤}⊗{\left(U^{\left(t-1\right)}\right)}^{⊤}⊗\cdots ⊗{\left(U^{\left(1\right)}\right)}^{⊤}\right)}^{⊤}{\left(G_{\left(t\right)}\right)}^{⊤}\\ = & G_{\left(t\right)}\left({\left(U^{\left(T\right)}\right)}^{⊤}U^{\left(T\right)}⊗\cdots ⊗{\left(U^{\left(t+1\right)}\right)}^{⊤}U^{\left(t+1\right)}⊗{\left(U^{\left(t-1\right)}\right)}^{⊤}U^{\left(t-1\right)}⊗\cdots ⊗{\left(U^{\left(1\right)}\right)}^{⊤}U^{\left(1\right)}\right){\left(G_{\left(t\right)}\right)}^{⊤}\\ = & {\left[\;\left[G;{\left(U^{\left(1\right)}\right)}^{⊤}U^{\left(1\right)},\cdots ,{\left(U^{\left(t-1\right)}\right)}^{⊤}U^{\left(t-1\right)},{\left(U^{\left(t+1\right)}\right)}^{⊤}U^{\left(t+1\right)},\cdots ,{\left(U^{\left(T\right)}\right)}^{⊤}U^{\left(T\right)}\right]\;\right]}_{\left(t\right)}{\left(G_{\left(t\right)}\right)}^{⊤} \end{array} \end{array}$$ (22)

In this way, by Eqs (21) and (22), we can directly compute Eq (10) and update **U**^(*t*)^ without calculating $S_{\left(t\right)}$. In other words, we don’t need to compute the Kronecker products round by round. **Algorithm 1** gives the pseudo-code of STFClus.

**Algorithm 1**: STFClus (Sparse Tensor Factorization based Clustering for Heterogeneous Information Networks).

1. Input relationship tensor X, number of clusters *K*, initial guess for ${\left\{U^{\left(t\right)}\right\}}_{t=1}^{T}$ and G, and convergence threshold *ϵ*.

2. **repeat**

3. **for** *t* ← 1 **to** *T*:

4. Update **U**^(*t*)^ according to Eqs (10), (21) and (22);

5. Normalize **U**^(*t*)^ according to Eq (11);

6. **end for**

7. Update G according to Eq (20);

8. **until** ${\left\|X-〚G;U^{\left(1\right)},U^{\left(2\right)},\cdots ,U^{\left(T\right)}〛\right\|}_{F}^{2}\leq \epsilon$.

9. Output ${\left\{U^{\left(t\right)}\right\}}_{t=1}^{T}$ and G.

#### Initialization

In the STFClus algorithm, the initial guess of the core tensor and factor matrices have a large impact on the final result. The best method for the core tensor and factor matrices initialization may vary between given real-world datasets. In general, each mode of the input tensor has its own physical meaning, and each element of the input tensor represents a relationship among different modes of the tensor. The STFClus algorithm aims to cluster all modes of the input tensor simultaneously by utilizing these relationships. Each factor matrix is the cluster indication matrix for a mode of the input tensor, and the core tensor is the mixture coefficient among different factor matrices.

As with the cluster indication matrices, the factor matrices should meet the constraints stated in Eq (5). It is clear that a factor matrix satisfying all the constraints is not unique, and the strategies for initialization are diversified. Of course, we can use random initialization, as it is simple and rapid. However, random initialization may lead to an increase in the number of iterations or even result in an unacceptably slow convergence speed.

Therefore, We propose a feasible method for initialization of the factor matrices that is similar to the traditional K-means method, called the STFClus_initial. We first cluster each mode of the input tensor independently as the corresponding mode factor matrix initialization; subsequently, the core tensor can be determined uniquely using the factor matrices.

Since STFClus_initial works in a similar way for different modes of input tensors, we will simply describe the process for a single mode. Without loss of generality, we detail the use of STFClus_initial on the *t*th mode of the input tensor. It is known that the *t*th mode of the tensor represents the *t*th type of objects in the heterogeneous information network. STFClus_initial on the *t*th mode of the input tensor can then be formalized as: given the tensor X of the heterogeneous information network, we want to partition the *t*th mode of X into *K* clusters.

The key aspect of STFClus_initial is how it measures the similarity between different objects. We note that, in the sparse representation of the input tensor $M={\left[m_{1:}^{⊤},m_{2:}^{⊤},\cdots ,m_{J:}^{⊤}\right]}^{⊤}$, each row **m**_*j*:_ = [*m*_*j*_1__, *m*_*j*_2__, ⋯, *m*_*j*_*T*__], for *j* = 1, 2, ⋯, *J*, corresponds to a nonzero element in the tensor, which indicates the relationship between the corresponding objects. The *t*th component of each row corresponds to the object from *t*th type.

According to **M**, we can define the similarity of two different *t*th type of objects (such as $\upsilon_{a}^{t}$ and $\upsilon_{b}^{t}$) as follows:

$$\begin{array}{c} sim\left(\upsilon_{a}^{t},\upsilon_{b}^{t}\right)=\frac{sam\left(\left\{m_{j:}|m_{j_{t}}=a\right\},\left\{m_{j:}|m_{j_{t}}=b\right\},t\right)}{\left(T-1\right)\text{max}\left(\left|\left\{m_{j:}|m_{j_{t}}=a\right\}\right|,\left|\left\{m_{j:}|m_{j_{t}}=b\right\}\right|\right)} \end{array}$$ (23)

where the |•| denotes the cardinality of a set, and the function ***sam***(•) denotes the total number of the same components in corresponding columns (except the *t*th column) of two matrices. For two matrices $A\in R^{r_{1}\times l}$ and $B\in R^{r_{2}\times l}$ with the same number of columns, and a natural number *t* ≤ *l*, the function ***sam***(•) can be defined as:

$$\begin{array}{c} sam\left(A,B,t\right)=\sum_{\underset{i\neq t}{i=1}}^{l}\left|\left\{a_{r,i}|a_{r,i}\in A,r=1,2,\cdots ,r_{1}\right\}\cap \left\{b_{r,i}|b_{r,i}\in B,r=1,2,\cdots ,r_{2}\right\}\right| \end{array}$$

According to Eq (23) we can see that the similarity function holds three properties:

1. $0\leq sim(\upsilon_{a}^{t},\upsilon_{b}^{t})\leq 1$
2. $sim(\upsilon_{a}^{t},\upsilon_{a}^{t})=1$
3. $sim(\upsilon_{a}^{t},\upsilon_{b}^{t})=sim(\upsilon_{b}^{t},\upsilon_{a}^{t}),a\neq b$

We denote the *K* clusters as $\left\{O_{1}^{t},O_{2}^{t},\cdots ,O_{K}^{t}\right\}$. We can also define the similarity between an object and a cluster as the weighted sum of the similarity between the object and each object in the cluster, namely,

$$\begin{array}{c} sim\left(\upsilon_{i}^{t},O_{k}^{t}\right)=\sum_{\upsilon_{j}^{t}\in O_{k}^{t}}u_{jk}^{\left(t\right)}sim\left(\upsilon_{i}^{t},\upsilon_{j}^{t}\right) \end{array}$$ (24)

Thus the probability of an object belonging to the corresponding cluster can be calculated as:

$$\begin{array}{c} u_{ik}^{\left(t\right)}=\frac{sim\left(\upsilon_{i}^{t},O_{k}^{t}\right)}{\sum_{k^{\prime }=1}^{K}sim\left(\upsilon_{i}^{t},O_{k^{\prime }}^{t}\right)} \end{array}$$ (25)

Furthermore, STFClus_initial on the *t*th mode of input tensor can be summarized as follows:

- Step1: Choose *K* objects from *t*th type as the initial clusters $\left\{O_{1}^{t},O_{2}^{t},\cdots ,O_{K}^{t}\right\}$ randomly. Here, we require that the similarity between any two of the *K* objects should not be equal to one.
- Step2: Calculate $sim(\upsilon_{i}^{t},O_{k}^{t})$, for *i* = 1, 2, ⋯, *N*_*t*_ and *k* = 1, 2, ⋯, *K* according to Eqs (23) and (24).
- Step3: Calculate $u_{ik}^{\left(t\right)}$, for *i* = 1, 2, ⋯, *N*_*t*_ and *k* = 1, 2, ⋯, *K* according to Eq (25).
- Step4: Repeat Step2 and Step3 until **U**^(*t*)^ unchanged or the iteration number is larger than a predefined number *iterNum*.

In practice, the algorithm will converge in less than 3 iterations in most cases. Since the **U**^(*t*)^ is only the initial guess for STFClus, and it will be updated in STFClus, we can set *iterNum* = 2.

After obtaining the initialization of **U**^(*t*)^, for *t* = 1, 2, ⋯, *T*, the core tensor G is determined uniquely by the factor matrices. According to the objective function in Eq (5), we can get the core tensor as follows:

$$\begin{array}{c} G=[\;\left[X;{\left(U^{\left(1\right)}\right)}^{†},{\left(U^{\left(2\right)}\right)}^{†},\cdots ,{\left(U^{\left(T\right)}\right)}^{†}\right]\;] \end{array}$$ (26)

where the superscript ‘^†^’ specifies the Moore-Penrose pseudo-inverse. The last constraint in Eq (5) makes sure that **U**^(*t*)^ is full column rank, i.e., the columns of **U**^(*t*)^ are linearly independent. So the Moore-Penrose pseudo-inverse can be calculated as:

$$\begin{array}{c} {\left(U^{\left(t\right)}\right)}^{†}={\left({\left(U^{\left(t\right)}\right)}^{⊤}U^{\left(t\right)}\right)}^{-1}{\left(U^{\left(t\right)}\right)}^{⊤} \end{array}$$ (27)

The pseudo-code of STFClus_initial is given in **Algorithm 2**.

**Algorithm 2**: STFClus_initial (An initial algorithm for STFClus).

1. Input relationship tensor X, number of clusters *K*.

2. **for** *t* ← 1 **to** *T*:

3. **do**

4. Choose *K* objects as initial clusters $\left\{O_{1}^{t},O_{2}^{t},\cdots ,O_{K}^{t}\right\}$ randomly;

5. **while** any $sim(\upsilon \in O_{k_{1}}^{t},\upsilon^{\prime }\in O_{k_{2}}^{t})==1$

6. **repeat**

7. **for** *i* ← 1 **to** *N*_*t*_:

8. **for** *k* ← 1 **to** *K*:

9. Calculate $sim(\upsilon_{i}^{t},O_{k}^{t})$ according to Eqs (23) and (24)

10. **end for**

11. Calculate $u_{ik}^{\left(t\right)}$ according to Eq (25)

12. **end for**

13. **until** **U**^(*t*)^ unchanged or *iterNum* > 2

14. **end for**

15. Calculate G according to Eqs (26) and (27);

16. Output the initial guess for ${\left\{U^{\left(t\right)}\right\}}_{t=1}^{T}$ and G.

#### Time complexity analysis

The time complexity for the proposed method comprises of two parts: STFClus_initial and STFClus. First, in STFClus_initial, we need to calculate the initial guess for factor matrices and core tensor. For the factor matrices initialization, we need to compute the similarity between each non-zero element in the tensor, i.e., the relationships in the heterogeneous information network, with each relationship containing *T* objects. So the time complexity for factor matrices initialization is **O**(*TJ*^2^), where *J* is the number of non-zero elements in the tensor. For the core tensor initialization, according to Eqs (26) and (27), we need to compute the Moore-Penrose pseudo-inverse of each factor matrix, and mode-n matrix product of tensor X with all factor matrices. The time complexity for computing the Moore-Penrose pseudo-inverse of all factor matrices is **O**(2*K*^2^ *N* + *TK*^3^), where $N=\sum_{t=1}^{T}N_{t}$ is the total number of objects in the network. So the time complexity for the core tensor initialization is **O**(*TKJ* + 2*K*^2^ *N* + *TK*^3^). Therefore, the total time complexity for STFClus_initial is **O**(*TJ*^2^ + *TKJ* + 2*K*^2^ *N* + *TK*^3^).

Second, in STFClus, we need to update the factor matrices and core tensor at each round. According to Eqs (21) and (22), computing $X_{\left(t\right)}S_{\left(t\right)}^{⊤}$ costs **O**((*T* − 1)*KJ* + *K*^*T*^ *N*_*t*_), and computing $U^{\left(t\right)}S_{\left(t\right)}S_{\left(t\right)}^{⊤}$ costs **O**(*K*^2^ *N* + *TK*^*T*+1^). So, the time complexity for updating all the factor matrices at each round is **O**((*T*^2^ − *T*)*KJ* + (*K*^*T*^ + *TK*^2^ + 3*K*)*N* + *T*^2^ *K*^*T*+1^). According to Eq (20), the time complexity for updating core tensor is **O**(*TKJ* + *K*^2^ *N* + *TK*^*T*+1^ + 2*K*^*T*^). Then, the total time complexity for STFClus is **O**(*T*^2^ *KJ* + (*K*^*T*^ + (*T*+1)*K*^2^ + 3*K*)*N* + (*T*^2^ + *T*)*K*^*T* + 1^ + 2*K*^*T*^).

For heterogeneous information networks, *T* is the number of object types, *K* is the number of clusters, *J* is the number of relationships and *N* is the total number of objects. We have *T* ≪ *J*, *T* ≪ *N*, and *K* ≪ *J*, *K* ≪ *N*. In order to show this more clearly, the time complexity for STFClus_initial can be summarized as **O**(*a*_1_*J*^2^ + *a*_2_*J* + *a*_3_*N* + *a*_4_), and the time complexity for STFClus can be summarized as **O**(*b*_1_*J* + *b*_2_*N* + *b*_3_), where *a*_1_, *a*_2_, *a*_3_, *a*_4_, *b*_1_, *b*_2_, and *b*_3_ are all constants. Thus, we can see that the time complexity for STFClus_initial is proportional to the number of objects and to the square of the number of relationships in the network, while the time complexity for STFClus is almost a linear function of the number of objects and relationships in the network.

## Experiments and results

In this section, we present several experiments on synthetic and real-world datasets for heterogeneous information networks, and compare the performance of our method, STFClus, with a number of state-of-the-art clustering methods.

All experiments are implemented in the MATLAB R2015a (version 8.5.0) 64-bit. The synthetic datasets are generated by the codes of synthetic datasets generation algorithm, which are shown in the S1 File. The real-world datasets are all publicly available online. The Matlab codes for STFClus_initial algorithm and STFClus algorithm are shown in the S2 File and the S3 File respectively. All the source codes are available online at https://github.com/tianshuilideyu/STFClus. The MATLAB Tensor Toolbox (version 2.6, http://www.sandia.gov/~tgkolda/TensorToolbox/) is used in our experiments. All experimental results are average values obtained by running the algorithms ten times on corresponding datasets, thus providing significant insight into the performance of different parameters and different algorithms.

### Dataset description

#### The synthetic datasets

The purpose of using synthetic datasets is to be able to verify the level of the performance that STFClus can deliver given that the detailed cluster structures of the synthetic datasets are known and so it is possible to evaluate the performance quantitatively based on the STFClus with different parameters.

The synthetic datasets are generated with the following parameters:

- *T*: the number of object types in the heterogeneous information network. It is also the number of modes in the tensor.
- *K*: the number of clusters.
- *S*: the tensor scale, and *S* = *N*_1_ × *N*_2_ × ⋯ × *N*_*T*_.
- *D*: the density of the tensor, i.e., the percentage of nonzero elements in the tensor. And $D=\frac{J}{S}$, where *J* is the number of nonzero elements.
- *O*: Whether the clusters are overlapping, denoted by a 1(yes) or 0 (no).

In order to make the synthetic datasets similar to a realistic situation, we assume the distribution for different types of objects that appear in a relationship to follow Zipf’s law (see details https://en.wikipedia.org/wiki/Zipf%27s_law). Zipf’s law is defined by $f_{t}\left(r;\rho_{t},N_{t}\right)=\frac{r^{-\rho_{t}}}{\sum_{n=1}^{N_{t}}n^{-\rho_{t}}}$, where *N*_*t*_ is the number of the *t*th type of objects, *r* is the object index, and *ρ*_*t*_ is the parameter characterizing the distribution. Zipf’s law denotes the frequency of the *r*th object of *t*th type appearing in the relationship. Then, with the parameters above, we can construct different synthetic datasets for different experiments.

**Experiment A** on synthetic datasets: in order to evaluate the performance quantitatively with different *D* and *S*, we fix *T* = 4, *K* = 2, and *O* = 1, and we set the parameter *ρ*_1_ = 0.95, *ρ*_2_ = 1.01, *ρ*_3_ = 0.99, and *ρ*_4_ = 1.05. We then construct four different scaled datasets, with *S* = 2.5*K*, *S* = 250*K*, *S* = 2.5*M* and *S* = 25*M*, respectively. For each network, we set different densities as *D* = 0.5%, *D* = 1%, *D* = 5% and *D* = 10% respectively. See details in Table 1.

**Table 1:** The synthetic datasets for Experiment A.

	*N*_1_ × *N*_2_ × ⋯ × *N*_*T*_	*D*
**Syn_a1**	10 × 5 × 5 × 10 = 2.5*K*	0.5%, 1%, 5%, 10%
**Syn_a2**	50 × 10 × 10 × 50 = 250*K*	0.5%, 1%, 5%, 10%
**Syn_a3**	50 × 10 × 50 × 100 = 2.5*M*	0.5%, 1%, 5%, 10%
**Syn_a4**	100 × 50 × 50 × 100 = 25*M*	0.5%, 1%, 5%, 10%

**Experiment B** on synthetic datasets: In order to evaluate the performance quantitatively with different *T* and *O*, we fix *K* = 2, *D* = 0.5% and *S* = 5*M*, and we set the parameter *ρ*_1_ = 0.95, *ρ*_2_ = 1.01, *ρ*_3_ = 0.99, *ρ*_4_ = 1.05, *ρ*_5_ = 0.9, *ρ*_6_ = 1.1, *ρ*_7_ = 0.95 and *ρ*_8_ = 1.05. We then construct four datasets with the same scale, in which *T* = 2, *T* = 4, *T* = 6 and *T* = 8 respectively, and for each *T*, we set *O* = 1 and *O* = 0 respectively. See details in Table 2.

**Table 2:** The synthetic datasets for Experiment B.

	*T*	*N*_1_ × *N*_2_ × ⋯ × *N*_*T*_	*O*
**Syn_b1**	2	5*K* × 1*K*	1, 0
**Syn_b2**	4	50 × 10 × 100 × 100	1, 0
**Syn_b3**	6	50 × 10 × 10 × 10 × 10 × 10	1, 0
**Syn_b4**	8	5 × 4 × 5 × 5 × 10 × 10 × 10 × 10	1, 0

#### The real-world datasets

In order to test the performance of STFClus in real-world scenarios, one medium-scale real-world dataset and two large-scale real-world datasets are used, and the details are summarized in Table 3.

**Table 3:** The details of real-world datasets.

	Types of objects	Number of objects	Number of relationships	Density
**DBLP-four-areas**	Author	14,475	334,832	9.01935 × 10^−9^
	Paper	14,376		
	Conference	20		
	Term	8,920		
**DBLP-full-areas**	Author	952,214	35,204,622	1.00896 × 10^−13^
	Paper	1,237,709		
	Venue	1,534		
	Term	192,995		
**Douban Movie Network**	Movie	12,677	441,008,031	1.20416 × 10^−16^
	Actor	6,311		
	Director	2,449		
	Type	38		
	User	13,367		
	Group	2,753		
	User_copy	13,367		

The first real-world dataset is extracted from the DBLP database, called DBLP-four-areas dataset, which contains the ground truth of cluster labels for some objects. It is a four research areas subset of DBLP used in [3–6, 8, 13, 36], and it can be downloaded from: http://web.cs.ucla.edu/~yzsun/data/DBLP_four_area.zip. The four research areas in the DBLP-four-areas dataset are database (DB), data mining (DM), machine learning (ML), and information retrieval (IR), respectively. There are five representative conferences in each area. All the related authors, the papers published in these conferences and the terms contained in the papers’ titles are included. The DBLP-four-areas dataset contains 14,376 papers with 100 labelled, 14,475 authors with 4,057 labelled, 20 labelled conferences and 8,920 terms. Here, there are no labelled records in terms, since terms are difficult to label even manually. In DBLP, many terms are included in multiple research areas, for example, ‘system’ is a high-frequency term in both DB and IR, and it also often appears in DM and ML. The frequencies of ‘system’ appearing in DB, DM, ML and IR are 31.65%, 23.10%, 10.41% and 34.83%, respectively. The density of the DBLP-four-areas dataset is 9.01935 × 10^−9^, so we can construct a medium-scale 4-mode tensor with size 14,376 × 14,475 × 20 × 8,920 and 334,832 non-zero elements. Each non-zero element in the 4-mode tensor represents a relationship or a sub-network in the DBLP, i.e., one author wrote a paper published on a conference and that contained a specific term. We compare the performance of STFClus with several other state-of-the-art methods on the labelled records in this dataset.

The second real world dataset is the DBLP database(downloaded form http://dblp.uni-trier.de/xml/ in August 2015), called DBLP-full-areas dataset, which contains all the research areas in computer science. It includes four types of objects: Author, Paper, Venue (conferences or journals) and Term, which are organized in a star network schema, as shown in Fig 1A. In the DBLP database, papers may come from journals, conferences, books, web pages and so on. We choose journal and conference papers in our experiment, because most journal and conference papers comprise the latest research results. Even so, the DBLP-full-areas dataset is still a large-scale dataset, containing 952,214 authors, 1,237,709 papers, 1,534 venues and 192,995 terms. The density of DBLP-full-areas dataset is 1.00896 × 10^−13^, so we construct a large-scale 4-mode tensor with size 952,214 × 1,237,709 × 1,534 × 192,995 and 35,204,622 non-zero elements. Compared with the DBLP-four-areas dataset, we can see that the increased number of multiple modes leads to an explosion of relationships (non-zero elements) in the generated tensor, although it is still very sparse.

For an additional case study, we use the Douban Movie Network, which is collected by Chuan Shi [37], and can be downloaded from https://github.com/zzqsmall/SemRec/tree/master/data. The Douban Movie Network follows a general network schema, as shown in Fig 1B, and includes 12,677 movies, 6,311 actors, 2,449 directors, 38 movie types, 13,367 users and 2,753 user groups. In addition to the attribute information of users and movies, the Douban Movie Network also includes social relations among users and recommendation actions between users and movies. The records of users, movies, directors and actors in this dataset are anonymous. In order to meet the restrictions of a heterogeneous information network in our work, we take a copy of users and denote it as user_copy, and organize the seven types of objects as the network schema shown in Fig 2. The density of the Douban Movie Network is 1.20416 × 10^−16^, so we construct a very large-scale7-mode tensor with size 12,677 × 6,311 × 2,449 × 38 × 13,367 × 2,753 × 13,367, and 441,008,031 non-zero elements. Each non-zero element in this 7-mode tensor represents a user with social relation information recommended a movie with the attribute information.

### Evaluation metrics

In order to compare the clustering results with other state-of-the-art clustering methods for heterogeneous information networks, we adopt the Normalized Mutual Information (NMI) [38] and Accuracy (AC) as our performance measurements.

NMI is used to measure the mutual dependence information between the clustering result and the ground truth. Given *N* objects, *K* clusters, one clustering result, and the ground truth classes for the objects, let *n*(*i*, *j*), *i*, *j* = 1, 2, ⋯, *K* be the number of objects that labelled *i* in clustering result while in the *j*th class of ground truth. The joint distribution can be defined as $p\left(i,j\right)=\frac{n(i,j)}{N}$, the marginal distribution of rows can be calculated as $p_{1}\left(j\right)=\sum_{i=1}^{K}p\left(i,j\right)$, and the marginal distribution of column can be calculated as $p_{2}\left(i\right)=\sum_{j=1}^{K}p\left(i,j\right)$. Then, the NMI is defined as:

$$\begin{array}{c} NMI=\frac{\sum_{i=1}^{K}\sum_{j=1}^{K}p\left(i,j\right)\text{log}\left(\frac{p(i,j)}{p_{1}\left(j\right)p_{2}\left(i\right)}\right)}{\sqrt{\sum_{j=1}^{K}p_{1}\left(j\right)\text{log}p_{1}\left(j\right)\sum_{i=1}^{K}p_{2}\left(i\right)\text{log}p_{2}\left(i\right)}} \end{array}$$

The NMI ranges from 0 to 1, the larger value of NMI, the better the clustering result is.

AC is used to compute the clustering accuracy that measures the percent of the correct clustering result. AC is defined as:

$$\begin{array}{c} AC=\frac{\sum_{t=1}^{T}\sum_{n=1}^{N_{t}}\delta \left(map\left(\upsilon_{n}^{t}\right),label\left(\upsilon_{n}^{t}\right)\right)}{\sum_{t=1}^{T}N_{t}} \end{array}$$

where $map(\upsilon_{n}^{t})$ is the cluster label of the object $\upsilon_{n}^{t}$; the $label(\upsilon_{n}^{t})$ is the ground truth class of the object $\upsilon_{n}^{t}$. The **δ**(⋅) is an indicator function:

$$\begin{array}{c} \delta \left(\cdot \right)=\{\begin{array}{ll} 1 & \text{if }map\left(\upsilon_{n}^{t}\right)=label\left(\upsilon_{n}^{t}\right)\\ 0 & \text{if }map\left(\upsilon_{n}^{t}\right)\neq label\left(\upsilon_{n}^{t}\right) \end{array} \end{array}$$

Since both of NMI and AC are used to measure the performance of clustering one type of object, the weighted average NMI and AC is also used to measure the performance of STFClus and other state-of-the-art methods:

$$\begin{array}{c} \bar{NMI}=\frac{\sum_{t=1}^{T}N_{t}{\left(NMI\right)}_{t}}{\sum_{t=1}^{T}N_{t}} \end{array}$$

$$\begin{array}{c} \bar{AC}=\frac{\sum_{t=1}^{T}N_{t}{\left(AC\right)}_{t}}{\sum_{t=1}^{T}N_{t}} \end{array}$$

### Experimental results

#### STFClus on synthetic datasets

**Fig 4:**
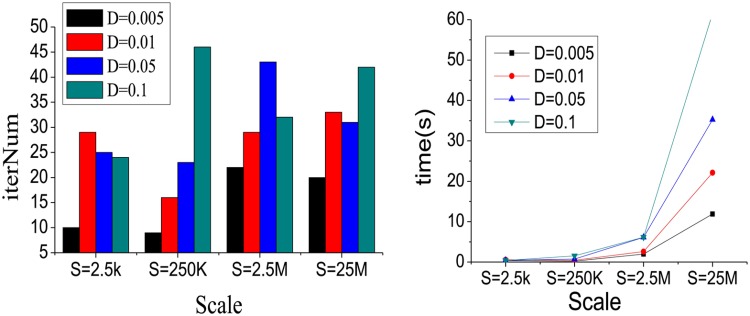
The iteration number and running time with different *D* and *S*.

**Fig 5:**
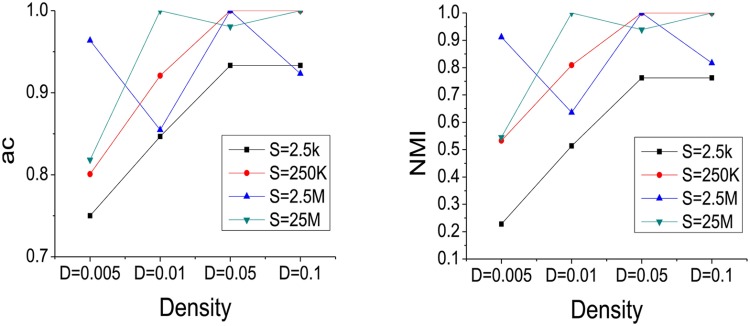
The AC and NMI values with different *D* and *S*.

**Experiment A**: In order to evaluate the performance quantitatively with different densities *D* and network scales *S*, STFClus is tested on the datasets in Table 1. Since there are four different densities for each scale network, the 16 synthetic datasets are grouped into 4 different scales networks. The experimental results are shown in Figs 4 and 5.

Fig 4 shows the iteration number and running time of STFClus on the synthetic datasets in Table 1. It should be noted that, since the running time of STFClus_initial algorithm on Syn_a4 with *D* = 5% and *D* = 10% is unacceptable, we use the random initialization method to initialize factor matrices on Syn_a4 with *D* = 5% and *D* = 10%. We also find that the STFClus doesn’t converge sporadically starting with the random initialization. In fact, non-convergence occurs two or three times out of ten. In addition, the iteration number and running time of STFClus are increased with increased network scale and density.

Fig 5 shows the AC and NMI of STFClus on the synthetic datasets in Table 1. We can find that with increased density, both AC and NMI are increased and become close to 1. This means that with the increase in network density, useful relationships in the network become richer and richer, and the clustering results become more and more close to the real world. When *D* = 0.5%, both AC and NMI on four synthetic datasets are low, since too few useful relationships exist in the network. Generally, larger network scales and density result in greater iteration numbers and running times, but offer higher accuracy and quality of clustering results.

To conclude, the use of synthetic networks in experiment A demonstrates that STFClus can work well on large-scale and sparse heterogeneous information networks.

**Fig 6:**
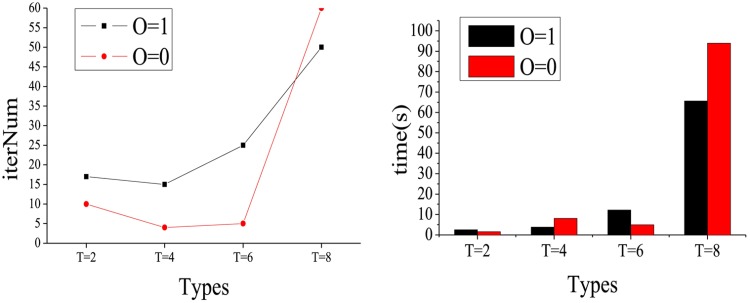
The iteration number and running time with different *T* and *O*.

**Fig 7:**
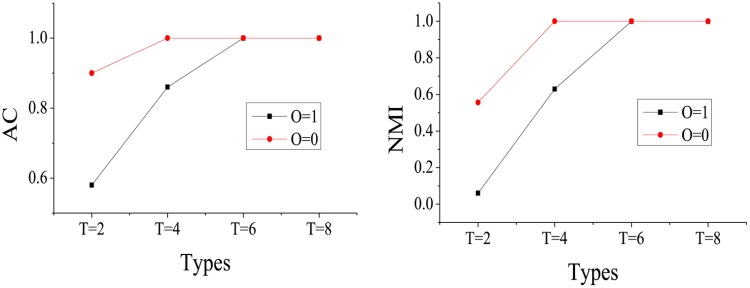
The AC and NMI with different *T* and *O*.

**Experiment B**: In order to evaluate the performance quantitatively with different object types *T* and various overlapping *O* states, we apply the STFClus method for the datasets in Table 2. In fact, there are 8 synthetic datasets grouped into 4 differently scaled networks, since each synthetic dataset has both overlapping and non-overlapping clusters. The experimental results are shown in Figs 6 and 7.

Fig 6 shows the iteration number and running time of STFClus on the synthetic datasets in Table 2. It can be found that with an increasing number of object types at the same network scale, the iteration number and running time are increased. When the clusters are non-overlapping, usually the iteration number is less than that when the clusters are overlapping. In Fig 6, both the iteration number and running time increase abruptly when *T* = 8. There are two possible reasons for this. First, the more object types *T* in the network, the more dimensions possessed by the tensor. This means that the number of factor matrices and the scale of core tensor would become larger when the object types *T* in the network is increased. The second reason is that when the network scale and density are fixed, the number of objects in each type, i.e., *N*_*t*_, is decreased while the object types *T* is increased. This phenomenon can be found in Table 2. When *T* = 8, *N*_*t*_ becomes less than ten. The network scale and density being fixed means that the non-zero elements in the tensor are unchanged. In other words, the number of relationships in the network remains unchanged while the network scale and density are fixed. With an increase in object types *T*, each relationship becomes more complex, i.e., each relationship contains more objects, and the frequency of each object appearing in the relationships is increased.

Fig 7 presents the AC and NMI results of the STFClus on synthetic datasets in Table 2. Both the AC and NMI are increased and equal to 1 with increasing number of object types. This means that when the network scale and density are fixed, the accuracy of the clustering results improves with increasing number of object types *T* in the network. We can also see that the clustering results of non-overlapping clusters are better when *T* = 2 and *T* = 4. However, the advantage disappears when *T* = 6 and *T* = 8. That is to say, when the number of object types *T* is small, the clustering results of STFClus on non-overlapping clusters are improved. However, when the number of object types *T* becomes sufficiently large, the clustering results of STFClus on both overlapping and non-overlapping networks are satisfactory. Because there are more object types in the network, more useful information about each object is shown through relationships.

Overall experiment B shows that STFClus can work better on networks with more object types. When the number of object types is sufficiently large, STFClus can handle networks with overlapping or non-overlapping clusters equally well.

#### STFClus on DBLP-four-areas dataset

In this section, the clustering performance of STFClus on the DBLP-four-areas dataset is compared with a number of state-of-the-art clustering methods as follows:

1. NetClus [3]: This is an extended version of RankClus [1], which can deal with networks following the star network schema.
2. PathSelClus [6, 8]: This is a clustering method based on the pre-defined symmetric meta-path, requiring user guidance. In PathSelClus, the distance between the same type object is measured by PathSim [5], and the method starts with seeds as given by the user.
3. FctClus [4]: This is a recently proposed clustering method for heterogeneous information networks. As with NetClus, the FctClus method can deal with networks following the star network schema.

As the baseline methods can only deal with heterogeneous information networks of a specific schema, here we must construct different sub-networks for them. For NetClus and FctClus, we use all four modes, but they are organized as a star network schema [3, 4], where the paper (P) is the centre type, and author (A), conference (C) and term (T) are the attribute types. For PathSelClus, we also use the four modes: author (A), paper (P), conference (C) and term (T). However, we select the symmetric meta-path of P-T-P, A-P-C-P-A and C-P-T-P-C to cluster the papers, authors and conferences respectively, and in PathSelClus, we give each cluster one seed to start.

Since the STFClus doesn’t need any information of network schema, we model the DBLP-four-areas dataset as a 4-mode tensor, and each mode represents one object type. The 4 modes are author (A), paper (P), conference (C) and term (T), respectively. The actual sequence of the object types is insignificant. Each element of the tensor represents a relationship among the four types of objects and we use the sparse representation of tensor. The AC, NMI and running time on DBLP-four-areas dataset of STFClus and the three baseline methods are summarized in Tables 4–6. From the experimental results on DBLP-four-areas dataset, we can see that STFClus performs the best on AC and NMI, while PathSelClus gives the best running time.

**Table 4:** AC of experiments on DBLP-four-areas dataset.

AC	STFClus	NetClus	PathSelClus	FctClus
**Paper**	0.7699	0.7154	0.7551	**0.7887**
**Author**	**0.8254**	0.7177	0.7951	0.8008
**Conference**	**0.9998**	0.9172	0.9950	0.9031
$\bar{AC}$	**0.8250**	0.7186	0.7951	0.8010

**Table 5:** NMI of experiments on DBLP-four-areas dataset.

NMI	STFClus	NetClus	PathSelClus	FctClus
**Paper**	0.7044	0.5402	0.6142	**0.7152**
**Author**	**0.8549**	0.5488	0.6770	0.6012
**Conference**	**0.9994**	0.8858	0.9906	0.8248
$\bar{NMI}$	**0.8520**	0.5503	0.6770	0.6050

**Table 6:** Running time of experiments on DBLP-four-areas dataset.

Running time (s)	STFClus	NetClus	PathSelClus	FctClus
**Paper**	—	802.6	**542.3**	808.4
**Author**	—	743.7	**681.1**	774.9
**Conference**	—	658.4	**629.3**	669.8
**Total time**	2840.9	2204.7	**1852.7**	2253.1

Though STFClus gives the longest running time in experiment, STFClus can obtain the clusters of all types of objects simultaneously, while the other baselines can only cluster one type of objects each time. This is why only the total time is shown for STFClus in Table 6. NetClus performs worse in the AC and NMI, just achieving 71.86% on AC and 55.03% on NMI. However, an important advantage of NetClus is that the objects ranking in each cluster can be obtained while clustering the objects. PathSelClus performs better than NetClus on AC and NMI. And it has an advantage too, i.e., based on the PathSim [5], PathSelClus can rapidly measure the similarity between any two objects of the same type using the predefined symmetric meta-path. PathSelClus also delivers the best result for running time. However, the results of PathSelClus strongly depend on the choice of meta-path and seeds as given by users.

#### Case studies on DBLP-full-areas dataset and Douban Movie Network

Since there is no ground truth for cluster labels of the DBLP-full-areas dataset and the Douban Movie Network, we cannot adopt AC and NMI to measure the performance of STFClus. In Table 3, we can see that the tensors constructed from both the DBLP-full-areas dataset and the Douban Movie Network are large-scale but low density. If we don’t use the sparse representation, the scale of the entire tensors may reach exabyte, or even zetabyte levels. Such large scale tensors are currently unrealistic for memory access and retrieval. Further, the storage of the entire tensor is unacceptable to most PCs. Although the storage space can be reduced to gigabyte (even megabyte) levels by using sparse representation and the computation of Kronecker products is avoided in STFClus, the intermediate results during the tensor decomposition may be much larger than the final result, and thus can lead to memory overflows.

To resolve such issues arising with larger-scale operations, we adopt the method introduced in [39] to divide the tensors constructed from both the DBLP-full-areas dataset and the Douban Movie Network into a grid of multiple smaller-scale sub-tensors and thereafter the STFClus is applied to all sub-tensors, and the results are re-constructed for the original tensors. In the experiment, Matlab Distributed Computing Server toolbox and Parallel Computing toolbox are used. All the experiments are run on a parallel system with 8 labs.

For DBLP-full-areas dataset, we divided the tensor with size 952,214 × 1,237,709 × 1,534 × 192,995 into a 1,000 × 1,000 × 100 × 1,000 dimensional grid that consists of 10^1^1 sub-tensors. We find that more than 99.98% of sub-tensors are zero tensors, i.e., all elements in these sub-tensors are zero elements. In practice, we maintain only the sparse sub-tensors, whose elements are not all zero elements, and their corresponding indices in the grid. Then, STFClus runs on all the sparse sub-tensors, whose elements are not all zero elements, simultaneously. For the sub-tensors whose elements are all zero elements, we set the elements in corresponding factor matrices and core tensors equal to zero. Finally, the strategy of re-constructing factor matrices and core tensor for original tensors in [39] is used. The same method is used to deal with the Douban Movie Network. We divided the tensor with size 12,677 × 6,311 × 2,449 × 38 × 13,367 × 2,753 × 13,367 into a 100 × 10 × 10 × 1 × 100 × 10 × 100 dimensional grid consisting of 10^9^ sub-tensors. More than 97.54% sub-tensors are zero tensors.

We set the number of clusters *K* = 15 for DBLP-full-areas dataset and *K* = 20 for Douban Movie Network. The details of implementation and results are summarized in Table 7. In Table 7, the non-zero sub-tensor represents the elements of sub-tensor that are not all zero elements.

**Table 7:** Case studies on DBLP-full-areas dataset and Douban Movie Network.

		DBLP-full-areas dataset	Douban Movie Network
**Non-zero sub-tensors**	number	15,437,462	24,623,145
	Max density	6.7225 × 10^−8^	4.5421 × 10^−10^
	Average density	6.7083 × 10^−10^	5.0024 × 10^−15^
**Running time (s)**	Grid generation	94,275	109,018
	Parallel computing of STFClus	65,641	54,412
	Factor matrices and core tensor reconstruction	44,548	87,521
	Total time	204,461	250,951

From Table 7, we can see that the number of non-zero sub-tensors is very large, although most sub-tensors are zero tensors in such a large-scale dataset. Moreover, all the non-zero sub-tensors are very sparse. The total running time includes three constituents: grid generation, parallel computing of STFClus, and factor matrices and core tensor reconstruction. For the DBLP-full-areas dataset and the Douban Movie Network, the grid generation and factor matrices and core tensor reconstruction took up most of the running time, while the parallel computing of STFClus just cost 32.1% of the time on the DBLP-full-areas dataset and 21.68% of the time on the Douban Movie Network. The system in total spent about 2.5 days to handle the DBLP-full-areas dataset and almost 3 days to handle the Douban Movie Network.

### Discussion

The experimental results on both synthetic and real-world datasets show that STFClus is an effective and efficient method for clustering heterogeneous information networks. It can handle all types of objects simultaneously, and obtain a good clustering result without any information on the network schema. In the experiments, we found that the random initialization of STFClus may lead to the non-convergence. That is to say, a good initialization can improve the performance of STFClus, and the STFClus_initial algorithm can provide a good start for STFClus.

Unfortunately, the current STFClus_initial algorithm is not perfect. It is highly efficient for sparse networks but not for dense networks. In other words, when the scale and density of the heterogeneous information network becomes large, the time cost of the STFClus_initial algorithm increases rapidly. In general, the network scale is usually large in real world applications, so STFClus_initial algorithm performs better with amaller the network density. We must thus make a compromise between the time complexity and efficiency of the whole method and this is a trade-off to be optimized by users on case-specific basis.

However, case studies on two very large-scale datasets show that STFClus can be used to analyze very large heterogeneous information networks off-line. The running time is acceptable, and STFClus has demonstrated high accuracy clustering results which can be used as prior knowledge for on-line analysis.

## Conclusions

Many clustering methods for heterogeneous information networks have been proposed, such as FctClus [4], NetClus [3], PathSelClus [6, 8] and so on. Each of them can deal with one type of heterogeneous information networks with a specified network schema. However, for general network schemas or in cases without any information of network schema, these clustering methods are not useable. Tensor factorization is a powerful tool for clustering multi-dimensional data. It has been widely used in some specific applications, such as computer graphics [16] and vision [40]. However, many existing tensor factorization based clustering methods focus on 3-mode tensors and clustering one mode of the tensor. In this paper, the STFClus method is presented as a way to cluster heterogeneous information networks based on sparse tensor factorization. The STFClus models heterogeneous information networks as a sparse tensor. In this approach, each type of objects in the network was modeled as one dimension of the tensor, and the relationships among different types of objects were modeled as the elements in the tensor.

In contrast to the existing clustering methods for heterogeneous information networks, STFClus has two distinct advantages. Firstly, STFClus can model different types of objects and the semantic relations in heterogeneous information networks without any information regarding the network schema. In addition, based on the tensor factorization, STFClus can cluster all types of objects simultaneously by running the algorithm only once; i.e. STFClus is generally applicable single-pass clustering method for heterogeneous network which is network schema agnostic.

Furthermore, an initialization algorithm is specifically developed for STFClus. In general, the initialization algorithm is good at handling sparse networks. The experimental results showed that STFClus can deal with large-scale and sparse heterogeneous information networks and perform better on networks with more types of objects. Moreover, STFClus can handle overlapping and non-overlapping clusters simultaneously. STFClus outperforms the state-of-the-art baselines on real-world datasets.

Nevertheless, STFClus is sensitive to the initialization of factor matrices and core tensor. A good initialization can improve the performance of STFClus, while a sub-optimal initialization may lead to an unacceptable slow convergence speed and unsatisfactory local minima. Although the STFClus_initial algorithm can provide a good initialization, the time cost increases rapidly for large scale and very dense networks.

In future work, this novel approach of clustering heterogeneous information networks based on tensor factorization can be combined with other rank-based clustering methods, e.g., RankClus and NetClus. Another challenge in future work is to deal with dynamically changing tensors as the heterogeneous information networks are changing. Possible solutions include increasing the number of tensor modes, or the number of dimensions of the tensor.

## Supporting information

S1 File The matlab codes of synthetic datasets generation algorithm. It uses Zipf’s law to generate the synthetic datasets for Experiment A and Experiment B.
(M) Click here for additional data file.

S2 File The matlab codes of STFClus_initial algorithm. (M)

S3 File The matlab codes of STFClus algorithm. (M)
